# Delayed gastric perforation following transarterial chemoembolization: Case report

**DOI:** 10.1016/j.ijscr.2023.107965

**Published:** 2023-03-04

**Authors:** Papawee Chennavasin, Jatuporn Phoopat, Preechapon Udomchaisakul, Montri Gururatsakul

**Affiliations:** a Department of Surgery, Princess Srisavangavadhana College of Medicine, Chulabhorn Royal Academy, Bangkok 10210, Thailand; b Division of Interventional Radiology, Department of Radiology, Princess Srisavangavadhana College of Medicine, Chulabhorn Royal Academy, Bangkok 10210, Thailand; c Department of Gastroenterology and Hepatology Chulabhorn Hospital, Princess Srisavangavadhana College of Medicine, Chulabhorn Royal Academy, Bangkok 10210, Thailand

**Keywords:** Transarterial chemoembolization, Gastric perforation, Ruptured hepatocellular carcinoma, Case report

## Abstract

**Introduction and importance:**

Transarterial chemoembolization (TACE) is widely employed to control acute bleeding in ruptured hepatocellular carcinoma (rHCC). Ischemia leading to perforation of the gastrointestinal tract (GIT) after TACE is a rare complication. We report a patient who presented with rHCC and who suffered gastric perforation post-TACE.

**Case report:**

A 70-year-old woman presented with rHCC. Emergency TACE was undertaken to control bleeding, and was successful. The patient was discharged 5 days post TACE. Two weeks after TACE, she presented with acute abdominal pain. Computed tomography of the abdomen showed perforation at the lesser curvature of the stomach. The angiogram from TACE was reviewed: the small vessels from an accessory branch of the left gastric artery originating from the left hepatic artery that had been embolized were likely responsible for gastric ischemia and subsequent perforation. The patient underwent operation with simple closure and omental patch repair. Postoperative gastric leak was not observed. Unfortunately, the patient died due to severe decompensated liver disease ∼4 weeks after TACE.

**Clinical discussion:**

Gastrointestinal tract (GIT) perforation after TACE is a rare complication. We suspected that perforation of the lesser curve of the stomach was secondary to ischemia due to non-target embolization to the accessory branch of the left gastric artery from the left hepatic artery, combined with stress and hemodynamic instability from rHCC.

**Conclusions:**

rHCC is a life-threatening condition. Variation in vascular structures must be clarified carefully. Significant adverse events in the GIT post-TACE are rare, but high-risk patients must be cautiously monitored.

## Highlights

• Variation in hepatic vascular structures must be clarified clearly during TACE/TAE.
• Major complications in the gastrointestinal tract following TACE are rare but potentially life-threatening because most patients have significant liver disease and a large tumor burden.
• High-risk patients such as rupture hepatoma should be monitored cautiously post-procedure.

## 1 Introduction [1]

The prevalence of ruptured hepatocellular carcinoma (rHCC) in Asian populations is up to 10 %–15 % [2]. rHCC is a life-threatening condition and associated with a poor prognosis, with a mortality rate of approximately 25 %–75 % [3]. In patients with HCC, liver failure is the third most likely cause of death, and accounts for 40 % of deaths [4].

Transarterial chemoembolization (TACE) or transarterial embolization (TAE) are employed widely to control acute bleeding due to rHCC. TACE and TAE are not highly invasive, are associated with low morbidity, and success can be achieved in 53 %–100 % of cases. Moreover, TACE/TAE can be used to treat hemodynamically unstable patients because these procedures can be carried out under local anesthesia. TACE can preserve liver function if a super-selective method of branch embolization is used [3].

Major complications from TACE can occur in ≤5 % of patients, and 1 % of patients may die after TACE [5]. TACE-associated complications can be divided into two categories: non-vascular and vascular-related. The most common non-vascular TACE-associated complication is postembolization syndrome. The prevalence of liver failure after TACE varies. In general, the risk of liver failure is dependent upon liver function and tumor burden at baseline. Other non-vascular TACE-related complications (*e.g.*, abscess, bacteremia, renal failure) are rare [2], [6]. TACE-associated vascular complications are usually associated with arterial access, such as hematoma, pseudoaneurysm, injury to the hepatic artery, and non-target embolization [5], [7].

Bleeding or perforation in the gastrointestinal tract (GIT) are rare complications following TACE. Few scholars have reported duodenal perforation or ischemia caused by non-target embolization. Ischemia of intraabdominal organs can also occur after several TACEs due to strictures and deformities of blood vessels. Stress-induced GIT ulcers have also been reported but, fortunately, most of them can be treated conservatively.

Here, we report delayed gastric perforation after TACE in a patient with rHCC. This clinical scenario has not been reported previously.

## 2 Presentation of case

A 70-year-old woman presented with acute abdominal pain and distension. Initially, computed tomography (CT) of the abdomen showed a background of cirrhosis, a hyper-vascular mass at segment 2/3 (size = 6 × 8 cm) with focal rupture and rim-enhancing fluid collection, which was likely to be rHCC. Another hyper-vascular mass was documented at segment 8 (size = 1 × 4 × 1 cm), which was likely to be HCC.

A liver screen was undertaken. The test for hepatitis B surface antigen was positive, so the initial diagnosis was chronic hepatitis-B virus infection associated with liver cirrhosis and HCC. The large HCC lesion at segment 2/3 was ruptured and causing abdominal pain and distension. She was hemodynamically stable at the time of presentation. She had no other significant medical history. At presentation, blood tests were undertaken for hemoglobin (8.8 g/dL), white blood cells (9000/μL), platelets (129,000/μL), International Normalized Ratio (1.24), bilirubin (0.63 mg/dL), albumin (3.0 g/dL), aspartate aminotransferase (83 U/L), alanine transaminase (33 U/L), alkaline phosphatase (78 U/L), and creatinine (1.1 mg/dL). She did not have antibodies against the hepatitis-C virus. She was classified as having Child–Pugh-A cirrhosis (compensated).

Urgent TACE *via* the left hepatic artery was carried out successfully. The left hepatic artery supplied the large tumor. Enucleation was observed at the left hepatic lobe (segment 2/3) and a small branch of the right hepatic artery, which supplied nodules to the right hepatic lobe (segment 7/8). Following TACE, the patient developed a hematoma at the right groin, which was managed conservatively. She was discharged from hospital 5 days after TACE.

**Fig. 1:**
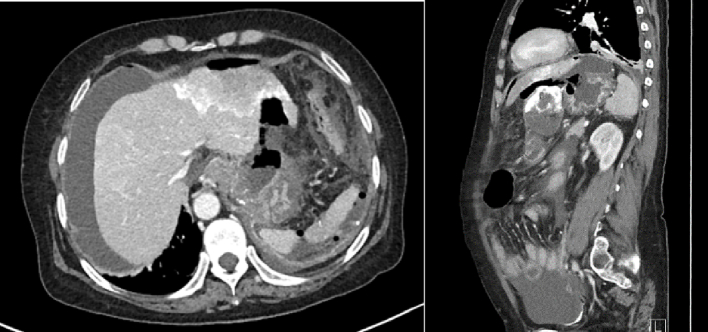
CT of the abdomen shows gastric-wall perforation and a previously concealed rHCC at the left hepatic lobe.

Two weeks after TACE, she presented at the emergency department of our hospital with sudden-onset abdominal pain of 2-h duration. Upon physical examination, vital signs were stable, the abdomen was distended, tenderness was noted in the epigastric area without guarding or rebound tenderness, and she had significant ascites. CT of the abdomen showed perforation at the lesser curvature of the gastric wall ∼2.2 cm in diameter with a small amount of pneumoperitoneum. A cirrhotic appearance was noted. Lipiodol staining revealed a reduced size (5.6 × 7.1 × 6.4 cm) of a previously concealed rHCC at the left hepatic lobe without a definite viable tumor (Fig. 1). Two partially lipiodol-stained nodules at hepatic segment VIII (size of 1.4 cm and 1.2 cm) without a definite viable tumor were newly observed. A moderate amount of ascites with newly observed lipiodol-stained moieties in the peri-splenic region and pelvic cavity were documented. A rim-enhancing hyperdense fluid collection (size = 1.7 × 3.2 × 6.0 cm) surrounded with fat reticulation at the right inguinal region was probably a hematoma.

**Fig. 2:**
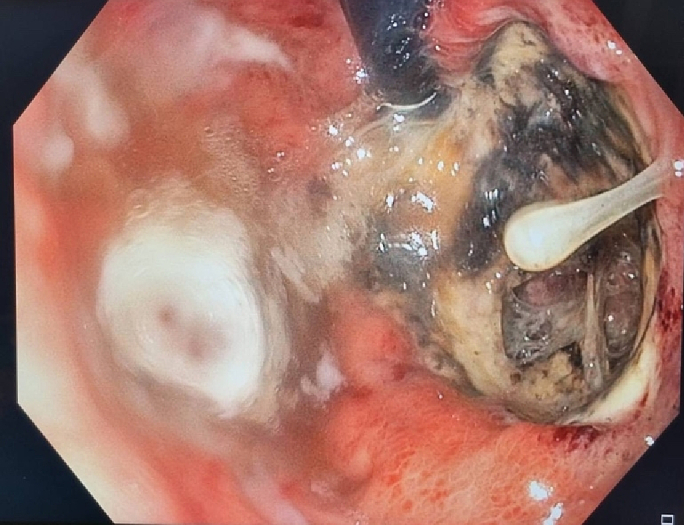
Intraoperative endoscopic finding; ischemic ulcer with a perforation at the lesser curve of the stomach.

**Fig. 3:**
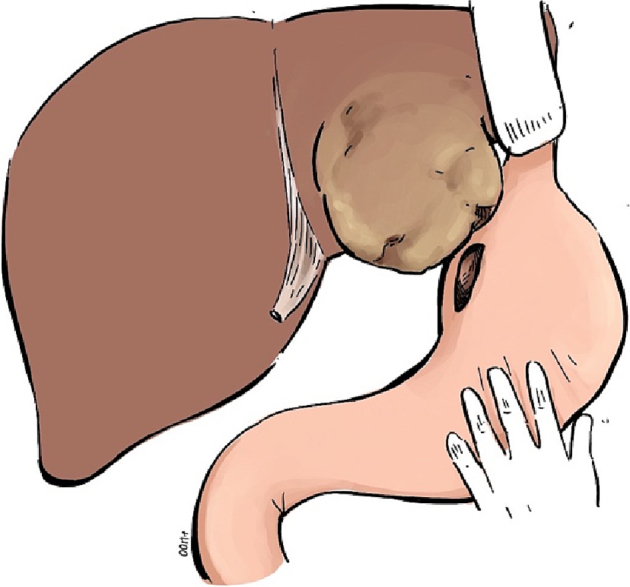
Operative finding: bulging HCC at the left liver lobe and a perforation 3 cm at the lesser curve of the gastric wall (schematic).

**Fig. 4:**
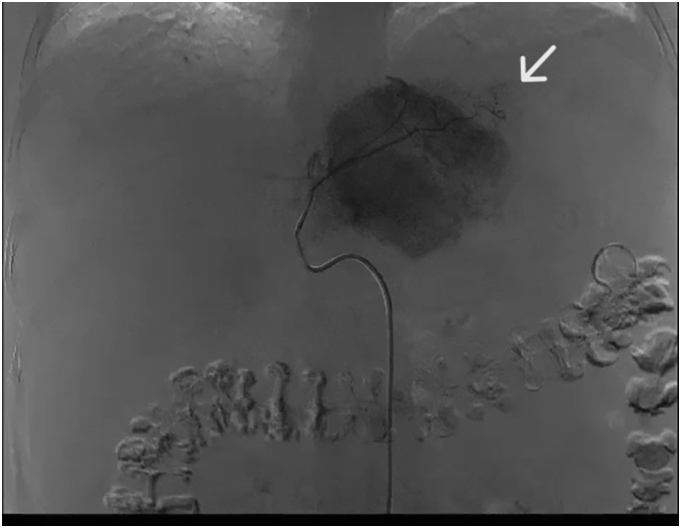
Angiogram: small vessels from an accessory branch of the left gastric artery originating from the left hepatic artery. (White arrow).

She was admitted to our hospital with gastric perforation. After appropriate initial treatment (intravenous hydration and broad-spectrum antibiotics), she was transferred to the operating theater for surgery and intraoperative esophagogastroscopy. Operative findings included an ischemic ulcer at the lesser curve of the stomach with a perforation site of size 3 cm (Fig. 2). Around 3 L of ascites with old blood were present. The left lobe of the liver had a bulging HCC with a necrotic part, but active bleeding was absent (Fig. 3). An area of gastric necrosis was debrided followed by simple closure and omental patch repair. A feeding jejunostomy tube was inserted. The angiogram from the initial TACE (Fig. 4) was reviewed. Small vessels from an accessory branch of the left gastric artery originating from the left hepatic artery that had been embolized were likely responsible for the ischemia and perforation of the gastric wall.

Postoperative leakage was not observed. The patient could tolerate enteral feeding *via* a jejunostomy tube and a liquid diet *via* the oral route. However, she developed decompensated cirrhosis (clinical and biochemical), and died 25 days after surgery due to progression of liver decompensation.

## 3 Discussion

In general, rHCC is associated with high mortality (0–37 %) [3]. TAE/TACE are safe and effective for controlling bleeding from acute rHCC. A perforation in the GIT secondary to ischemia after TACE is a rare complication. Hirakawa and colleagues showed that 45 % (13/29) of patients who underwent TACE developed superficial gastrointestinal mucosal lesions (possibly due to ischemia and stress) but no patient developed perforation in the GIT [8]. Su and colleagues found that 65 % (16/26) of patients showed new gastroduodenal lesions upon endoscopy that were clinically insignificant [9]. Several studies have reported ischemia in the GIT after TAE/TACE, but most cases resulted from non-target embolization or because patients were undergoing multiple TACEs and developed vascular strictures [10], [11], [12]. Most of those patients presented with GIT bleeding or abdominal pain. Perforation in the GIT after TACE is rare, and only one study has reported duodenal perforation after multiple TACEs [13]. There is no available data in gastric perforation secondary to ischemia after TACE due to the rich vascular supply of stomach.

In our patient, we suspected that perforation of the lesser curve of the stomach was secondary to ischemia due to non-target embolization to the accessory branch of the left gastric artery from the left hepatic artery. Although, the non-target embolization may rarely cause perforation, since stomach have enriched blood supply, the endoscopic view found area of ischemia at lessor curve which was the area that supply by left gastric artery and the perforation site was in the middle of ischemic area. We suspected that this severe ischemia might be non-target embolization combined with stress and hemodynamic instability from rHCC.

## 4 Conclusions

rHCC is a life-threatening condition. Variation in vascular structures must be clarified clearly during TACE/TAE. Major complications in the GIT following TAE/TACE are rare but potentially life-threatening because most patients have significant liver disease and a large tumor burden. High-risk patients should be monitored cautiously post-procedure.

## Informed consent statement

Written informed consent was obtained from the patient and patient's next of kin (upon patient's death) for publication of this case report and accompanying images. A copy of the written consent is available for review by the Editor-in-Chief of this journal on request.

## Ethical approval

The authors declare that the work described has been carried out in accordance with the Declaration of Helsinki of the World Medical Association revised in 2013 for experiments involving humans. Ethical Approval was provided by the authors institution.

## Funding

None.

## Author contribution

Papawee Chennavasin : Study concept, Drafting of manuscript

Jatuporn Phoopat : Data collection

Preechapon Udomchaisakul : Data interpretation

Montri Gururatsakul : Critical revision of manuscript

## Guarantor

Papawee Chennavasin.

## Research registration number

Not application.

## Declaration of competing interest

This research did not receive any specific grant from funding agencies.
